# Atypical Presentation of a Preeclamptic Patient With Severe Features and Auditory Hallucinations

**DOI:** 10.7759/cureus.100264

**Published:** 2025-12-28

**Authors:** Nichole Cufino, Tania S Flink, Carlos Lamoutte

**Affiliations:** 1 College of Osteopathic Medicine, Lake Erie College of Osteopathic Medicine, Bradenton, USA; 2 College of Obstetrics and Gynecology, BayCare's South Florida Baptist Hospital, Plant City, USA

**Keywords:** auditory hallucinations, bipolar disorder, hypertension, hypertensive disorders of pregnancy, posterior reversible encephalopathy syndrome, preeclampsia

## Abstract

The primary purpose of the case study was to investigate an unusual case of preeclampsia (PreE) with severe features accompanied by atypical posterior reversible encephalopathy syndrome (PRES) and auditory hallucinations in the setting of a medical history of bipolar affective disorder. A 41-year-old, G2 P0 A1, morbidly obese (pregnancy body mass index = 42.8 kg/m²) white woman with a history of bipolar affective disorder and no previous prenatal care presented to the emergency room with a hypertensive crisis (262/138 mmHg) and complaints of headaches and visual changes for the past four days. Signs of proteinuria (dipstick reading: 3+) and renal insufficiency (creatinine: 1.9 mg/dL) were observed upon admission. Due to these factors, an urgent low transverse Cesarean section was performed. On post-operative day one, the patient continued to present with high blood pressure despite anti-hypertensive medications. Additionally, she reported auditory hallucinations, polyuria, blurry vision, and vomiting. A head computed tomography (CT) scan and magnetic resonance imaging (MRI) without contrast presented an atypical presentation of PRES with brainstem abnormalities and prominent white matter involvement found uniquely in the temporal lobes and bilateral hippocampal gyri. An additional hypertensive crisis (196/126 mmHg) occurred on post-operative day four, which was controlled with hydralazine. This case study highlights the need for close monitoring of patients with bipolar disorder and adequate prenatal care to avoid the adverse effects seen in hypertensive disorders of pregnancy. Additionally, the relationship between uncontrolled bipolar disorder and auditory hallucinations is explored.

## Introduction

Preeclampsia (PreE) is a hypertensive disorder of pregnancy that occurs after 20 weeks of gestation and is typically accompanied by proteinuria. The systolic blood pressure (SBP) must be greater than 140 mmHg or a diastolic blood pressure (DBP) greater than 90 mmHg recorded at two different times and four hours apart [1]. Additionally, severe PreE is diagnosed if the SBP is greater than 160 mmHg, or the DBP is greater than 110 mmHg, recorded twice at least four hours apart, which the patient had met criteria for on presentation. A protein dipstick reading of 2+ (300 mg) or greater from a 24-hour urine collection is also indicative of PreE; if not present, thrombocytopenia, impaired liver function, severe right upper quadrant (RUQ) pain, renal insufficiency, pulmonary edema, or new-onset headache that does not subside with medication or explained by other alternate diagnoses must be observed [1]. Furthermore, the diagnosis of eclampsia is made when there is an onset of tonic-clonic, focal, or multifocal seizures in women with hypertensive disorders of pregnancy (HDP) [1].

Posterior reversible encephalopathy syndrome (PRES) is also associated with PreE and more commonly with eclampsia. PRES consists of either vasogenic edema in the subcortical white matter, as seen in most patients, or cytotoxic edema [2]. Common neurologic symptoms with either an acute or subacute onset include headaches, visual changes, seizures, consciousness impairment, mental disorders, focal neurologic deficits, nausea, and vomiting [2]. The etiology of PRES is unknown; however, hypomagnesemia has been proposed as a contributing factor [3]. Hypomagnesemia is normally treated with magnesium sulfate, due to its ability to regulate vascular function, reduce inflammation, and decrease the permeability of the blood-brain barrier [3].

For this case report, an atypical presentation of PreE with severe features and PRES accompanied by a history of bipolar affective disorder was explored. The significance of this case was the unique presentation of PRES, which included uncontrolled hypertension and auditory hallucinations that cannot be interpreted by the bipolar diagnosis alone.

## Case presentation

Medical history

A 41-year-old multigravida white woman (G2 P0 A1) at 33 weeks of gestational age, estimated by emergency department ultrasound, with a lack of prenatal care, unmedicated chronic hypertension, and advanced maternal age, presented to the emergency department with a chief complaint of headache and visual changes for four days. The patient’s medical history additionally included an unknown last menstrual period, unmedicated bipolar affective disorder, and Class III Morbid Obesity with a body mass index (BMI) of 42.8 kg/m^2^. Her obstetric history included a 19-week intrauterine fetal demise. Her documented medications included 100 mg bupropion by mouth (PO), thrice per day (TID), alprazolam (0.5 mg PRN or as needed) for anxiety, melatonin (PRN for sleep), pantoprazole (40 mg, PO, once per day, or QD), and phentermine (37.5 mg, PO, QD), but she reported not having the medication for about one week. Additionally, the patient denied taking bupropion or Cymbalta (dosage and frequency unknown) for the past seven months, after becoming unemployed in November.

Clinical features

Day 1

**Figure 1:**
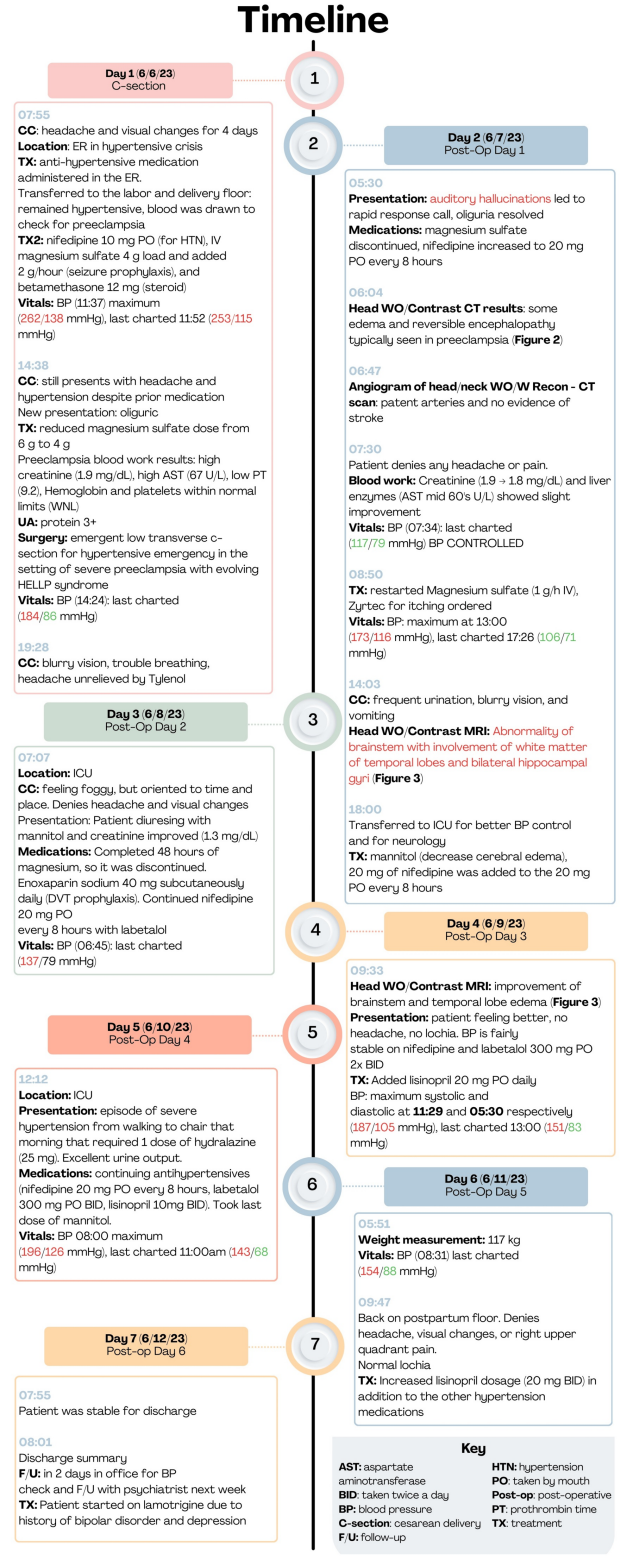
Visual timeline of clinical course and management over a seven-day hospitalization. Note: DVT - deep venous thrombosis; HELLP - hemolysis, elevated liver enzymes, low platelets; RUQ - right upper quadrant; SQ - subcutaneous; WO/W - without or with reconstruction

A condensed timeline of the clinical features observed in this patient can be found in Figure 1. In addition, a chronology of laboratory tests from days one to six is presented in Table 1. On June 6, 2023, the patient presented to the emergency department at 8 AM with a maximum blood pressure of 262/138 mmHg, for which she was given nifedipine (10 mg PO) to control the hypertensive emergency. The ultrasound fetal emergency protocol was conducted due to vaginal bleeding and suspected PreE and presented an estimated 33-week-old fetus. She continued to be hypertensive at 184/86 mmHg despite being given three anti-hypertensive medications. She was then admitted and transferred to the labor and delivery floor to be started on IV magnesium sulfate (4 g load and added 2 g/hour) for seizure prophylaxis, Celestone (12 mg), and other confirmatory tests for preeclampsia. The blood work was positive for a high red blood cell distribution width (RDW) of 15.4, possibly for compensation of hemolysis, high liver enzymes with aspartate aminotransferase (AST) of 67 U/L, normal platelets at 220 x 109/L, and signs of renal insufficiency with a high creatinine of 1.9 mg/dL and proteinuria with dipstick reading of 3+.

**Table 1:** Chronology of laboratory findings from day one to day six Note: RDW = red blood cell distribution width; AST = aspartate aminotransferase liver enzyme; ALT = alanine aminotransferase liver enzyme

Lab Test	Day 1	Day 2	Day 3	Day 4	Day 5	Day 6	Reference range
Blood pressure (mmHg)	262/138 (max); 184/86 (with meds)	117/79 (with nifedipine)	137/79	187/105 (max)	196/126 (with hydralazine)	154/88	< 120/80
Hemoglobin (g/dL)	14.8	13	10.3			9.4	12-15.5
RDW (%)	15.4					17.2	11.5-14.5
AST (U/L)	67	65	41				5-34
ALT (U/L)	38	40	29				0-55
Prothrombin Time (s)	9.2	9.1					9.8-13.3
Fibrinogen (mg/dL)	579	477					198-468
Platelets (x10^9^/L)		220	149			228	140-450
Creatinine (mg/dL)	1.9	1.8	1.3				0.6-1.1
Urine Protein (qualitative)	3+						Negative

Later, at about 3 PM, the patient became oliguric and had an unremitting headache despite Tylenol and magnesium administration. An urgent low transverse Cesarean section (C-section) was performed due to her unstable presentation of PreE with severe features, which included severe blood pressure ranges and persistent headache despite medical treatment and elevated liver enzymes, indicating early hemolysis, elevated liver enzyme levels, and low platelet levels (HELLP) syndrome development. The infant after delivery was reportedly healthy with an APGAR score of 8 and 9 and a Ballard score, showing a 37-week-old infant, in contrast with 33 weeks as previously estimated in the ER. After the C-section at about 7:30 PM, she reported blurry vision, trouble breathing, and a continued headache unrelieved by Tylenol.

Day 2

**Figure 2:**
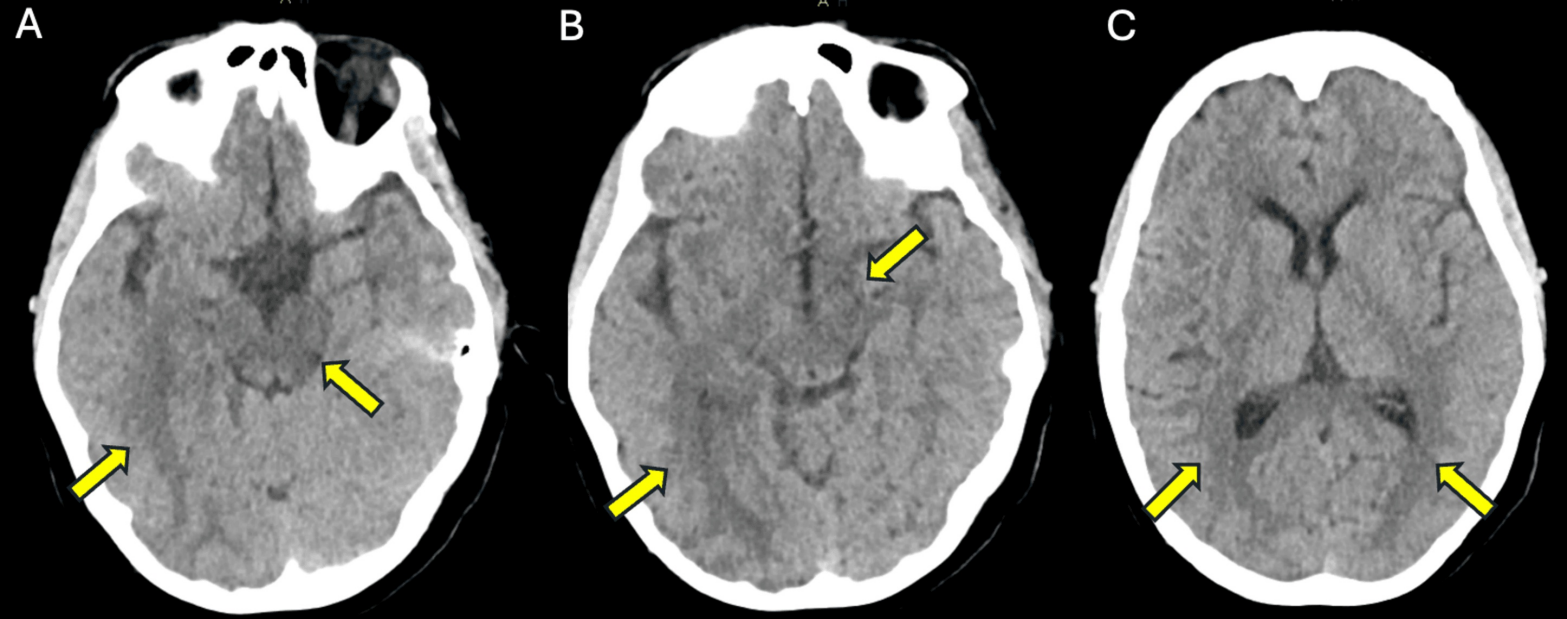
Head CT without contrast showing edema from 6/7 Note: Panels A, B, and C from June 7 progress from caudal to cephalad views, respectively, to show hippocampal and brainstem edema (yellow arrows). No repeat CT scans were performed on June 9.

**Figure 3:**
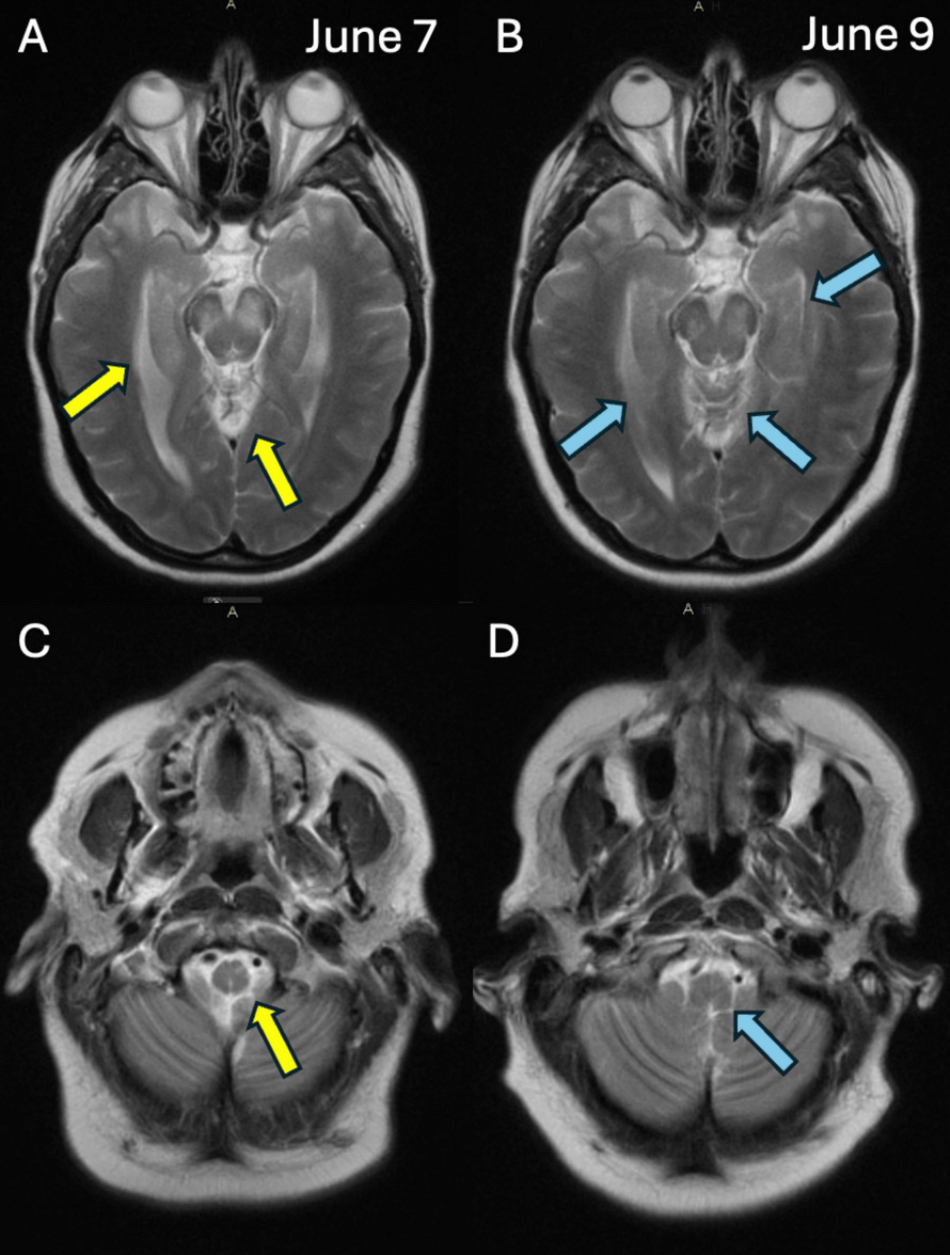
Head MRI edema changes from 6/7 to 6/9 Note: Panels A and C (June 7) show hippocampal and brainstem edema (yellow arrows). Panels B and D (June 9) show improvements in edema (blue arrows).

On June 7, 2023, at approximately 5:30 AM, the patient reported auditory hallucinations, which led to a rapid response call, and imaging of the head and neck was ordered. However, upon the review of systems, the patient was alert and oriented x4 and with no reported findings of pressurized speech, grandiosity, or rapid flight of ideas. Intraoperative and post-operative oliguria were resolved, but blood pressure continued to increase. Nifedipine was increased to 20 mg every eight hours. A head CT scan without contrast showed expansion of the brainstem, which possibly contributed to underlying edema and PRES, typically seen in PreE (see also Figure 2). The angiogram of the head/neck with and without contrast and reconstruction showed patent arteries and no evidence of stroke. Additionally, a magnetic resonance imaging (MRI) without contrast of the head MRI was performed and showed an abnormality of the brainstem with involvement of the white matter of the temporal lobes and bilateral hippocampal gyri (see Figure 3, panels A and C). The MRI report stated that these findings could be an atypical presentation of PRES and the presence of white matter disease representing ischemic demyelination that was out of proportion for her age.

Magnesium sulfate was discontinued after the onset of auditory hallucinations due to fear of magnesium toxicity, but there were no signs of respiratory depression or loss of deep tendon reflexes. After consulting neurology, magnesium was restarted for 24 hours, at which time the patient denied headache or pain. Blood work showed a coagulation panel within normal limits, a hemoglobin of 13 g/dL, creatinine had now decreased to 1.8 mg/dL, and liver enzymes also began to decrease to the mid-60 U/L levels. Blood pressure was controlled for the first time at 117/79 mmHg with nifedipine 20 mg every eight hours, labetalol 300 mg twice per day (BID), and lisinopril 20 mg BID. At about 6 PM, neurology advised to keep tighter blood pressure control due to brain MRI showing brainstem edema, so the patient was transferred to the ICU, and mannitol was added along with 20 mg of nifedipine to the already prescribed 20 mg nifedipine every eight hours. 

Day 3

On June 8, the patient reported feeling foggy, but she was oriented to time and place. She denied having a headache and visual changes, and she completed 48 hours of magnesium, which was then discontinued. Lovenox 40 mg subcutaneous daily was given for deep vein thrombosis prophylaxis, and nifedipine 20 mg PO every eight hours was continued with labetalol. Blood pressure increased to 137/79 mmHg, but the patient was diuretic with mannitol, and creatinine improved to 1.3 mg/dL. The patient was also seen by psychiatry, where her diagnosis of bipolar affective disorder and discontinued use of bupropion and Cymbalta for seven months after unemployment was clarified. The patient also denied any impulsivity and presented with appropriate behavior and insight.

Day 4

On day four, June 9, blood pressure was moderately stable on the nifedipine, labetalol, and lisinopril 10 mg BID, which was added in the ICU. The blood pressure maximum was 187/105 mmHg, but the patient overall felt better and denied headache or lochia. A repeat MRI scan of the head without contrast showed improvement of the brainstem and temporal lobe edema (see Figure 3, panels B and D).

Day 5

On June 10, the patient continued to take the antihypertensives in the ICU. She had a severe episode of hypertension with her blood pressure rising to 196/126 mmHg. This occurred when she stood up to walk to her chair in the morning; this was remedied with one dose of hydralazine 25 mg IV. Additionally, she took her last dose of mannitol and had excellent urine output.

Day 6

On June 11, the patient was back on the post-partum floor and denied headache, visual changes, or right upper quadrant pain. The patient also had normal lochia, weight was recorded at 117 kg, and her blood pressure was measured at 154/88 mmHg. The clinical goal was to keep her systolic blood pressure below 150 mmHg, so the lisinopril dosage was increased to 20 mg BID. Otherwise, the patient was stable for discharge.

Day 7

On June 12 (post-operative day six), behavioral health was examined, and the patient denied suicidal or homicidal ideation and reported having a good support system. She was discharged with a recommended follow-up with the OB/GYN in two days for a blood pressure check. She was provided with lamotrigine 25 mg QD for two weeks and one tablet by mouth twice weekly. Because of this patient’s history of bipolar affective disorder and depression, lamotrigine was prescribed, with a follow-up with a psychiatrist the following week. The patient was also discharged home with nifedipine 10 mg PO, TID; labetalol 300 mg, BID; lisinopril 20 mg, BID; and medications for pain relief as needed. By day seven, her auditory hallucinations had resolved, and the patient was stable for discharge on triple oral antihypertensive therapy and lamotrigine for bipolar affective disorder.

## Discussion

The patient in this case initially presented to the emergency room with hypertension, headache, visual changes, and 3+ urine proteinuria, which were suggestive of PreE. After her C-section was performed to treat the PreE, the patient experienced auditory hallucinations on day two of admission. The continuation of hypertension, blurry vision, and headache unrelieved by Tylenol post C-section were suggestive of PRES; however, the overall presentation was atypical in this case. For one, the patient experienced a hypertensive crisis (262/138 mmHg) beyond the typical range observed in practice, which continued despite the use of three anti-hypertensive medications. Additionally, PreE with severe features included a risk of ischemic or hemorrhagic stroke [4] and seizure onset, as seizures are observed in up to 87% of patients with PRES [2].

The patient experienced visual changes for four days. A systematic review with 387 reported cases of PRES found that it typically presents white matter changes in the occipital lobe [5]. The most frequent reported involvement of the brain was in the occipital lobe (84.2%), followed by parietal (67.9%), and frontal area (32.2%) in a total of 387 cases. In addition to visual changes, she also experienced polyuria, blurred vision, and vomiting, which manifested post-operatively on day two. Blurred vision is a common complaint, along with increased risk of blindness, hypertensive retinopathy, exudative retinal detachment, and cortical blindness [6]. Hence, these presentations are typically observed in PRES presentations.

The atypical presentation is in the auditory hallucinations, which manifested on day two, and were unaccompanied by an altered mental status or symptoms aligned with a manic episode. The presence of auditory hallucinations in an otherwise alert and oriented patient made a stronger argument against other possible differential diagnoses, such as delirium, primary psychosis, or an exacerbation of bipolar disorder. In addition, the MRI presented an atypical presentation of PRES with an abnormality of the brainstem along with white matter involvement of the temporal lobes and bilateral hippocampal gyri. The current literature does not report auditory hallucinations as a commonly reported feature of PreE; because neurological complications typically occur in only 20% of preeclamptic patients [3], the general presence of neurological symptoms already places this patient in a small category of patients, and her unforeseen presentation makes her case most likely the first to be reported in the literature.

Two theories are proposed on the mechanisms behind the presentation of auditory hallucinations in PRES. For one, the temporal lobe of the brain may be more vulnerable to hypertensive states due to its partial arterial supply from the posterior circulation of the brain. The posteromedial temporal lobes are supplied by the posterior circulation of the brain via the P3 segment of the posterior cerebral artery [7]. Compared to the anterior circulation of the brain, the posterior circulation has less sympathetic reflex control and autoregulation to counter the vasodilatory effects of the parasympathetic innervation, which may result in uncontrolled hyperperfusion [2]. Although there are currently several ongoing theories of the pathophysiology of PRES, this hyperperfusion may lead to the vasogenic edema found in PRES [8]. Therefore, this lack of autoregulation in the temporal lobe can explain its increased susceptibility to hypertensive states, leading to abnormal sensory experiences.

Second, the patient’s medical history and lack of prenatal care may have contributed to her auditory hallucinations. Inadequate prenatal care is associated with poor obstetric outcomes, which include preterm delivery, PreE, and stillbirth [9]. In addition, adverse pregnancy outcomes, including HDP and PreE, are frequently observed in patients with bipolar disorder [10]. Preventing HDP in pregnancy is important due to the increased risk of future maternal myocardial infarction, heart failure, chronic hypertension, and stroke [11]. Therefore, care for these patients can be expanded with an early referral to high-risk obstetrics, blood pressure monitoring at home, and appropriate visits with a multidisciplinary care team, including the fields of OB/GYN, psychiatry, internal medicine, and social work. Additionally, early psychiatry referral and pharmacologic management of bipolar disorder beyond initial therapy should be individualized according to maternal risk factors, with a thorough assessment of potential risks and benefits during pregnancy.

While the use of bupropion to control the patient’s bipolar affective disorder may have contributed to the onset of auditory hallucinations, it is less likely in this patient as she denied taking bupropion for seven months prior to emergency room admission for PreE. Enhanced dopamine levels of the brain following bupropion administration may induce psychosis and perceptual changes [12]. Bupropion also decreases the threshold of developing a seizure and increases the risk of seizure development [2,13]. However, despite the increased risk, the patient did not progress to an eclamptic state since she never developed a seizure. It is suggested that early hypertension and magnesium management may have played a role in preventing the onset of a seizure.

Key takeaway points are as follows: (1) high-risk patients with severe PreE and bipolar affective disorder may develop PRES with atypical imaging and symptom patterns; (2) patients with appropriate obstetric high-risk factors, such as Class III Morbid Obesity or history of fetal demise, are recommended to be consulted by maternal-fetal medicine for pregnancy management; (3) auditory hallucinations in this context should prompt urgent neuroimaging and multidisciplinary assessment (neurology, psychiatry, and obstetrics); (4) inadequate prenatal care and suboptimal psychiatric management in early pregnancy increases the risk of severe hypertensive and potential neurologic complications; and (5) guidelines on proper bipolar disorder management in pregnancy, including risks and benefits of medication, should be considered.

## Conclusions

This current study examined an atypical presentation of PRES characterized by temporal lobe and hippocampal involvement with associated auditory hallucinations in the setting of PreE and notably without the progression to an eclamptic episode despite common risk factors. In addition, the patient’s medical history of bipolar affective disorder, persistent hypertensive crisis despite treatment, and onset of auditory hallucinations present a unique manifestation of severe PreE. This case highlights the need for appropriate prenatal care and close monitoring of high-risk patients diagnosed with bipolar disorder to avoid potential adverse effects observed in hypertensive disorders of pregnancy.
